# Supplementary material for: Outcomes of Endoscopic Endonasal Dacryocystorhinostomy in Glaucoma Patients
Source: J Pers Med. 2024 Mar 27;14(4):348. doi: 10.3390/jpm14040348 (PMC11050967; doi:10.3390/jpm14040348)
Supplement: Supplementary file 1 [file jpm-14-00348-s001.zip › jpm-2928291-supplementary.pdf]

Table S1. Survey

		Left eye	Right eye
	Grade		
Epiphora	0	No epiphora	No epiphora
	1	Epiphora requiring dabbing ≤ twice/day	Epiphora requiring dabbing ≤ twice/day
	2	Epiphora requiring dabbing 2-4 times/day	Epiphora requiring dabbing 2-4 times/day
	3	Epiphora requiring dabbing 5-10 times/day	Epiphora requiring dabbing 5-10 times/day
	4	Epiphora requiring dabbing > 10 times/day	Epiphora requiring dabbing > 10 times/day
Dacryocystitis	#6	No	No
	#7	Occasionally (< one/month)	Occasionally (< one/month)
	#8	Often (> one/month)	Often (> one/month)
